# Investigating possible causal effects of externalizing behaviors on tobacco initiation: A Mendelian randomization analysis

**DOI:** 10.1016/j.drugalcdep.2018.07.015

**Published:** 2018-10-01

**Authors:** Meg E. Fluharty, Hannah Sallis, Marcus R. Munafò

**Affiliations:** a MRC Integrative Epidemiology Unit (IEU) at the University of Bristol, United Kingdom; b UK Centre for Tobacco and Alcohol Studies, School of Experimental Psychology, University of Bristol, United Kingdom; c Centre of Academic Mental Health, Population Health Sciences, Bristol Medical School, University of Bristol, United Kingdom

**Keywords:** Externalizing behavior, Tobacco use, Mendelian randomization

## Abstract

Observational studies suggest childhood externalizing disorders are associated with increased smoking and earlier initiation. However, causality cannot be inferred from observational data alone. The current study uses two-sample MR to examine the causal relationship between externalizing behaviors and tobacco use.

Single nucleotide polymorphisms (SNPs) associated with aggression were obtained from the Early Life Epidemiology Consortium (mean age 8), ADHD from the Integrative Psychiatric Research and Psychiatric Genomics Consortiums (age range 6–18), and tobacco initiation and age of onset from the Tobacco and Genetics Consortium. SNPs were combined using the inverse variance weighted approach, weighted median approach, and MR-Egger regression.

There was no clear evidence of an effect of aggression on tobacco initiation or age of onset for childhood aggression (initiation: β −0.002, 95% CI −0.005, 0.001, P = 0.286; age: β −0.001 95% CI −0.002, 0.000, P = 0.310) or adolescent aggression (initiation: β −0.001, 95% CI −0.006, 0.003, P = 0.610; age: β 0.000, 95% CI 0.000, 0.001, P = 0.183)]. However, there was some evidence of an association of ADHD on tobacco initiation (OR 1.23, 95% CI 1.10, 1.35, P = 0.016), although no clear evidence of an effect of ADHD on age of onset (OR = 1.022, 95% CI 0.992, 1.052, P = 0.215).

Our results provide some evidence that genetic risk of childhood ADHD is causally related to increased risk of tobacco initiation; however, the causal estimate is relatively small. We found no clear evidence that genetic risk of childhood aggression is causally related to the risk of tobacco initiation or age of onset.

## Highlights

• There’s some evidence genetic risk of ADHD is causally related to tobacco initiation.
• There’s no clear evidence genetic risk of childhood aggression is causally with tobacco use.
• Multiple Mendelian randomization methods were used to strengthen causal inference.

## 1 Introduction

Externalizing disorders such as conduct disorder (CD) and attention-deficit hyperactivity disorder (ADHD) are characterized by behaviors including aggressiveness, impulsivity, sensation seeking, and often criminal behavior at older ages (Brazil et al., 2016; Goldstein et al., 2017; Holmes et al., 2001). Externalizing disorders are strongly associated with substance use, including tobacco use (Brook et al., 2014; Kim et al., 2009; Brook et al., 2010), and externalizing behaviors or symptoms below a clinical threshold are similarly associated with increased risk of smoking (Kollins et al., 2005). Individuals with externalizing disorders have similar rates of smoking as other psychiatric disorders (∼>40%), experience higher rates of smoking, earlier onset of smoking, and greater nicotine dependency (Pomerleau et al., 1995; John et al., 2004; Lasser et al., 2000; Bagot et al., 2007).

Externalizing disorders are associated with deficits in reward function – reduced sensitivity to favorable stimuli and increased reward sensitivity. (Verdejo-Garcia et al., 2008). For example, ADHD is characterized by abnormal functioning of the striatal dopaminergic system and disruptions in dopaminergic transmission within corticostriatal circuits, resulting in executive functioning deficits (McClernon and Kollins, 2008; Solanto, 1998). These individuals display decreased dopamine (DA) tone from below-normal presynaptic activation of DA autoreceptors resulting in exaggerated DA release to salient stimuli (McClernon and Kollins, 2008). In both clinical and preclinical studies, nicotine is shown to stimulate striatal DA release, suggesting nicotine-stimulated DA release may be more rewarding in individuals with ADHD (McClernon and Kollins, 2008; Corrigall et al., 1994; Brody et al., 2004). Therefore, individuals with ADHD may find higher levels of reward reinforcement from initial tobacco use, which may, in turn, facilitate the transition to continued use (McClernon and Kollins, 2008).

Additionally, externalizing disorders are characterized by impulsive behavior (behavior that lacks foresight, evaluation, and inhibitory control). These individuals typically prefer immediate rewards (over possible larger rewards in the future) (Verdejo-Garcia et al., 2008). Therefore, impulsivity may lead individuals to choose rewarding stimuli despite the possibility of negative outcomes. A hypersensitive reward system may predispose individuals to early-onset substance use or mediate the transition from experimental to more frequent/ daily use (Aklin et al., 2009). Animal studies have supported this hypothesis, as rodents divided into ‘high-impulsivity’ and ‘low impulsivity’ groups based on attentional tasks show differential response to drug self-administration. High-impulsive displayed more rapid acquisition and higher rates of drug self-administration in response to decreased levels of dopamine D2-receptor binding (Dalley et al., 2007). These findings are mirrored in human PET studies where decreased striatum D2-receptor density is observed in addicted individuals, resulting in increased positive psychological and physiological response to initial drug exposure (Volkow et al., 1993, 1996; Wang et al., 1996).

Tobacco may also be used for self-medication in individuals with ADHD to alleviate ADHD symptoms through negative reinforcement (Laucht et al., 2007; Rodriguez et al., 2008). Studies using transdermal nicotine patches alongside stimulant medicine have displayed increased attention capabilities in adult ADHD patients (Gehricke et al., 2006). Furthermore, studies using nicotine patches in non-smoking ADHD patients have displayed increases in attention and cognition similar to methylphenidate (Potter and Newhouse, 2004), improved behavioral inhabitation (Potter and Newhouse, 2004) and increased positive affect (Levin et al., 1996; Rodriguez et al., 2008; McClernon and Kollins, 2008).

However, the causal nature of this relationship and the direction of causality remain unclear. Evidence from longitudinal studies suggests that externalizing behaviors in children are associated with subsequent early onset tobacco use (Korhonen et al., 2010; Brook et al., 2010; Crone and Reijneveld, 2007; Audrain-McGovern et al., 2004), and while reverse causality is unlikely in this context due to the onset of externalizing disorders in young children (prior to average smoking initiation ∼age 15), residual confounding may still be operating (e.g. via intrauterine tobacco exposure). Experimental studies are typically impossible in substance use research for long-term outcomes for obvious ethical and practical reasons. While we can examine the association between externalizing behaviors and tobacco use in observational studies, these have provided conflicting evidence on the temporal direction of association (Korhonen et al., 2010; Cadoret et al., 1983; Brook et al., 2010; Crone and Reijneveld, 2007; Hicks et al., 2009). Ultimately, however, conventional epidemiological methods using observational data cannot support a strong causal inference as problems such as selection bias, reverse causation, and residual confounding is inherent in observational data (Davey Smith and Ebrahim, 2001). It is therefore important to consider alternative methods to support the stronger causal inference, such as the use of negative controls, cross-contextual designs, and instrumental variable analysis (Gage et al., 2016b). One type of instrumental variable analysis is Mendelian randomization (MR), in which genetic variants robustly are predicting an exposure of interest is used as an unconfounded proxy for that exposure. Therefore, any associations found between these genetic variants and the outcome provide evidence of a casual effect of the exposure captured by the genetic variants on the outcome, without the risk of reverse causation or confounding. Two-sample MR uses variant-exposure associations identified via genome-wide association studies (GWAS), and variant-outcome associations from another independent GWAS, using publicly-available summary data (Burgess et al., 2015). The current study uses two-sample MR to examine the causal relationship between externalizing behaviors and tobacco initiation (Bowden et al., 2015).To compare across the spectrum of externalizing disorders we used data available from both an aggression and an ADHD GWAS.

## 2 Methods

### 2.1 Exposure measures

For our externalizing behaviors exposure, we used summary data from the Early Life Epidemiology Consortium (EAGLE) consortium GWAS of aggressive behavior on a sample of 18,988 individuals. Aggression was measured using continuous study-specific scales (Pappa et al., 2016). Six SNPs were identified as the top SNPs approaching genome-wide significance (P < 5 × 10^−5^) for aggression. The GWAS further identified top SNPs stratified by age group, with five SNPs approaching genome-wide significance for early childhood (mean age 5.36 years, SD 1.5), and six SNPs for middle childhood/early adolescence (mean age 11.39 years, SD 1.86) (see Supplementary Table S1).

For our ADHD exposure, we used summary data from the Initiative for Integrative Psychiatric Research (iPSYCH) and Psychiatric Genomics Consortium (PGC) GWAS of ADHD on 55,354 individuals (ages 6–19) (Demontis et al., 2017). ADHD was measured using the binary cohort-specific diagnosis of ADHD. Fourteen independent SNPs (were identified as genome-wide significant (P < 5 × 10^−8^) (see Supplementary Table S2).

### 2.2 Outcome measures

For our tobacco initiation outcome, we used summary data from the Tobacco and Genetics Consortium (TAG) GWAS of smoking behavior (Tobacco and Genetics Consortium, 2010). Tobacco initiation (n = 74,053) was a binary ever/never measure and age of initiation (n = 24,114) was the reported age at which participants started smoking.

### 2.3 Statistical analyses

SNPs associated with aggression and ADHD were identified in their respective GWAS and subsequently extracted from the tobacco GWAS (see Supplementary Tables S1–S2). Where original SNPs were unavailable, proxy SNPs were identified using SNiPA (http://snipa.helmholtz-muenchen.de/snipa3/) with an r^2^ threshold of ≥0.9. SNP-exposure and SNP-outcome associations were combined using an inverse-variance weighted approach (IVW), weighted median approach, and MR-Egger regression. Here, we use multiple methods, each with differing underlying assumptions regarding instrument validity, to triangulate our results (Lawlor et al., 2016). IVW weights regression of SNP-exposure and SNP-outcome coefficients restricting the intercept to zero, and assumes all instruments are valid with no pleiotropy (Burgess et al., 2013). Weighed median provides a causal estimate if at least 50% of the instruments are valid (Mostafavi et al., 2016). MR-Egger uses an intercept coefficient in the weighted regression to relax the assumption that the outcome works strictly via the exposure (i.e., up to 100% of the instruments may be invalid). The intercept term displays the overall pleiotropic effect, while the slope (β) coefficient displays a causal estimate under the assumption the pleiotropic effects of the SNP on the outcome are unrelated to the associations between the SNP and exposure (Corbin et al., 2016). Finally, I^2^ statistics were calculated to determine heterogeneity between estimates from multiple genetic instruments, with higher numbers indicating greater heterogeneity which could indicate potential violations of the MR assumptions. Main findings are presented as IVW within the text. To avoid inference based simply on P-value thresholds, the direction and strength of effect for each association, together with the corresponding P-value, is presented (Sterne and Davey Smith, 2001). All analyses were conducted in R (version 3.3.2). Additionally, there was no direct overlap between samples. However, it is possible some parents in the TAG may have offspring included in EAGLE.

## 3 Results

### 3.1 Aggression and tobacco initiation

There was no clear evidence of an effect from the genetic risk of aggression to tobacco use initiation (β_IVW_<0.001, 95% CI −0.003 to 0.002, P = 0.810). While the MR-Egger analysis suggested there is some evidence of an effect on initiation, the effect estimates were inconsistent with those from the IVW and weighted median analyses (see Table 1 for full results). Findings were similar when looking at the effect from the genetic risk of aggression to age at onset, with no clear evidence of an effect observed (β_IVW_<0.001, 95% CI: 0.000–0.001, P = 0.125). These findings were consistent when restricted to early childhood and late adolescence (see Table 1). There was moderate to high heterogeneity (see Table 1 for full results).

**Table 1:** Estimates of the causal effects of the risk of aggression behaviors on tobacco initiation and age of onset using IVW, MR-Egger and weighted median Mendelian randomization approaches.

	Initiation			Age of onset		
Method	β	95% CI	*P* value	β	95% CI	*P* value
**Aggression (all ages, mean age = 8.4)**						
IVW	0.000	−0.003, 0.002	0.810	0.000	0.000, 0.001	0.125
Weighted median	0.001	−0.003, 0.002	0.759	0.000	0.000, 0.001	0.161
MR-Egger slope	−0.039	−0.056, −0.021	0.012	0.004	−0.001, 0.008	0.160
MR-Egger intercept	0.019	0.170, 0.204	0.012	−0.017	−0.038, 0.004	0.188
Heterogeneity	I^2^ = 36%					

**Aggression (early childhood, ages 3-7)**						
IVW	−0.002	−0.005, 0.001	0.286	−0.001	−0.002, 0.000	0.310
Weighted median	−0.003	−0.008, 0.003	0.388	0.000	−0.001, 0.001	0.578
MR-Egger slope	−0.014	−0.024, 0.004	0.066	−0.001	−0.006, 0.004	0.812
MR-Egger intercept	0.032	0.007, 0.058	0.088	0.000	−0.013, 0.014	0.966
Heterogeneity	I^2^ = 82%					

**Aggression (late adolescent, ages 8-15)**						
IVW	−0.001	−0.006, 0.003	0.610	0.000	0.000, 0.001	0.183
Weighted median	−0.001	−0.006, 0.003	0.572	0.000	0.000, 0.001	0.292
MR-Egger slope	−0.016	−0.005, 0.025	0.051	0.000	−0.002, 0.002	0.862
MR-Egger intercept	−0.069	−0.113, −0.025	0.037	0.001	−0.008, 0.009	0.878
Heterogeneity	I^2^ = 93%					

### 3.2 ADHD and tobacco initiation

There was weak evidence of an association from the genetic risk of ADHD to tobacco initiation (OR_IVW_ 1.23, 95% CI 1.10–1.35, P = 0.016), and low heterogeneity (I^2^  = 29%). Although MR-Egger analysis indicated that there was some evidence of pleiotropy (OR_Intercept_ 1.06, 95% CI 1.03–1.09, P = 0.020), the bias-adjusted effect estimate remained consistent with that estimated using the IVW approach and suggested some weak evidence of a causal effect (OR_Slope_ 1.40, 95% CI 1.10–1.78, P = 0.087). There was no clear evidence of an effect from the genetic risk of ADHD to the age of onset (see Table 2 for full results).

**Table 2:** Estimates of the causal effects of the risk of ADHD on tobacco initiation and age of onset using IVW, MR-Egger and weighted median Mendelian randomization approaches.

	Initiation			Age of onset		
Method	OR	95% CI	*P* value	OR	95% CI	*P* value
**ADHD**						
IVW	1.230	1.110, 1.350	0.016	1.022	0.992, 1.052	0.215
Weighted median	1.180	−1.033, 1.327	0.068	1.014	0.947, 1.081	0.705
MR-Egger slope	1.440	1.101, 1.779	0.087	0.928	0.797, 1.059	0.315
MR-Egger intercept	1.060	−1.027, 1.093	0.020	1.004	0.997, 1.011	0.202
Heterogeneity	I^2^ = 29%					

### 3.3 Power calculation

Post-hoc power calculations were conducted for our IVW analyses using an online Mendelian randomization power calculation tool (https://sb452.shinyapps.io/power/) (Burgess, 2014). For the analysis investigating the effect of aggression with tobacco use initiation, using a genetic instrument that explains 1% of the variation in aggression and a sample containing 28% smoking cases, our study has 77% power to detect a causal effect of β = 0.1. Power was similar when looking at the effect of aggression on age at onset. For the analysis investigating the effect of ADHD with tobacco use initiation, using a genetic instrument which explains 2% of the variation in ADHD and a sample containing 28% smoking cases, our study has 73% power to detect a causal effect of OR = 1.2. Power was similar when looking at the effect of ADHD on age at onset.

## 4 Discussion

Our results provide some evidence that genetic risk of childhood ADHD is causally related to increased risk of tobacco initiation; however, the causal estimate is relatively small. We found no clear evidence that genetic risk of childhood aggression is causally related to the risk of tobacco initiation or age of onset. Therefore, the observational evidence relating to these associations (Kretschmer et al., 2014; Disney et al., 1999; Korhonen et al., 2010; Rohde et al., 2004) may not be causal and may be due to environmental or confounding factors.

Our findings with respect to genetic risk of ADHD and tobacco use initiation correspond with observational evidence (Breyer et al., 2014; Milberger et al., 1997; Kollins et al., 2005; Lambert and Hartsough, 1998), and the converging evidence from different MR methodologies provides further confidence in these findings. Neurobiological evidence also supports these causal associations (McClernon et al., 2008). However, as the MR-Egger analyses suggest some evidence of horizontal pleiotropy, the use of alternative causal inference methods relying on different assumptions (e.g. cross-contextual comparisons across populations where confounding structures differ) will allow triangulation to determine whether these results do reflect true causal relationships (Gage et al., 2015; Munafo and Davey Smith, 2018).

Within individuals with ADHD, a hypoactive striatal dopaminergic system produces increased dopamine transporter within corticostriatal circuits. This subsequently decreases dopamine tone and activation of presynaptic autoreceptors, resulting in exaggerated dopamine response to salient stimuli. As nicotine stimulates a response in the striatum of smokers, nicotine-stimulated phasic dopamine releases may be more rewarding for individuals with ADHD (Dresel et al., 2000; Krause et al., 2002, 2003; Grace, 2001). Therefore, this heightened reward response to dopamine in individuals with ADHD may be responsible for reward and maintenance effects (Krause et al., 2000; Grace, 2001).

These findings may help identify individuals particularly vulnerable to tobacco use behaviors, and therefore provide interventions from an early age. For example, previous evidence indicates that strong familial bonds and open communication within families and schools may serve as a protective factor, or help to delay adolescent substance initiation (McArdle et al., 2002; Kliewer and Murrelle, 2007; Farrell and White, 1998; Spoth et al., 2004).

There are a number of important strengths to this study. The use of Mendelian randomization analysis strengthens causal inference and removes the possibility of reverse causation, confounding, and bias (Gage et al., 2015, 2016a). Furthermore, we integrated multiple methods with varying assumptions and strengths to formulate more robust causal inferences (Gage et al., 2015). The use of two-sample MR utilizes large sample sizes to provide the sufficient power required to detect small effects in complex phenotypes. However, there are also some limitations to consider. Unlike childhood aggression, which was a measurable trait of externalizing disorders, ADHD was only measured as a binary diagnosis. Previous evidence has suggested that particular traits of ADHD (i.e., inattention or impulsivity/hyperactivity) are more associated with the increased likelihood of tobacco initiation (McClernon et al., 2008). However, the current GWAS only examined ADHD diagnosis and not by individual symptomology. A GWAS of these individual traits has been conducted in adulthood (Mick et al., 2014), but sample sizes were small, and summary statistics were unavailable. Finally, we conducted several analyses, which raises the possibility that some of the effects we report may represent false positives. This should be considered when interpreting our results as a whole.

Overall our results provided some evidence that there may be a causal association of genetic risk of ADHD on tobacco initiation, these results are in line with previous longitudinal and neurobiological evidence. However, there was no association between early aggressive behavior on tobacco initiation, suggesting these observed associations may be a result of additional variables. Our results suggest that prevention efforts should target these risk groups, and explore whether interventions to reduce these behaviors influence subsequent tobacco use initiation. By identifying at-risk individuals, prevention behaviors such as strengthening familial bonds, open communication within families and schools, and early intervention education may help delay or prevent adolescent substance use initiation. (Steinberg et al., 1994; McArdle et al., 2002; Dishion et al., 2002; Kliewer and Murrelle, 2007; Farrell and White, 1998; Spoth et al., 2004).

## Role of funding source

None declared.

## Contributors

All authors designed the study. MEF conducted the analysis and drafted the manuscript with input from MRM and HS. All authors approved the final version of the manuscript for submission.

## Conflict of interests

No conflict declared.
