# Supplementary material for: Investigating possible causal effects of externalizing behaviors on tobacco initiation: A Mendelian randomization analysis
Source: Drug Alcohol Depend. 2018 Oct 1;191:338–42. doi: 10.1016/j.drugalcdep.2018.07.015 (PMC6152577; doi:10.1016/j.drugalcdep.2018.07.015)
Supplement: Supplementary file 1 [file mmc1.docx]

**Supplementary Material for the Article:**

Investigating possible causal effects of externalizing behaviors on tobacco initiation:

A Mendelian randomization analysis

**This material supplements, but does not replace, the peer-reviewed paper in**

***Drug and Alcohol Dependence*.**

Meg E Fluharty ^1,2^, Hannah Sallis ^1,2,3^, Marcus R Munafò ^1,2^

1. MRC Integrative Epidemiology Unit (IEU) at the University of Bristol, United Kingdom.
2. UK Centre for Tobacco and Alcohol Studies, School of Experimental Psychology, University of Bristol, United Kingdom.
3. Centre of Academic Mental Health, Population Health Sciences, Bristol Medical School, University of Bristol, United Kingdom.

**Correspondence:**

Meg Fluharty

School of Experimental Psychology, University of Bristol

12a Priory Road, Bristol BS8 1TU, United Kingdom

E-mail: meg.fluharty@bristol.ac.uk

**Table S1.** List of SNPs associated with aggression (P x10^-5^)

|  |  |  |  |  |  |
| --- | --- | --- | --- | --- | --- |
| **SNP** | | **Aggression** | | **Tobacco initiation** | |
|  |  | **β** | **SE** | **OR** | **SE** |
| rs10508552 | | 4.783 | 0.307030 | 1.000 | 0.0211 |
| rs11126630 | | 5.441 | 0.244876 | 0.978 | 0.0122 |
| rs2015436 | | 4.802 | 0.269508 | 0.983 | 0.0211 |
| rs2079515 | | 4.597 | 0.244198 | 1.009 | 0.0116 |
| rs7625357 | | 4.607 | 0.269819 | 1.013 | 0.0127 |
| rs9372149 | | 4.858 | 0.272832 | 1.030 | 0.0131 |
|  | | **Early Aggression** | | **Tobacco initiation** | |
| rs11760485 | | 2.954 | 0.261911 | 0.985 | 0.0127 |
| rs1577595 | | 1.779 | 0.268604 | 1.002 | 0.0128 |
| rs17086954 | | 2.384 | 0.261637 | 1.002 | 0.0129 |
| rs2763339 | | 3.344 | 0.254246 | 0.987 | 0.0122 |
| rs589804 | | 2.213 | 0.243537 | 1.007 | 0.0117 |
|  | | **Late Aggression** | | **Tobacco initiation** | |
| rs11126630 | | 5.441 | 0.244876 | 1.023 | 0.0122 |
| rs11700808 | | 3.339 | 0.248604 | 0.986 | 0.0124 |
| rs11977715 | | 2.420 | 0.303979 | 0.970 | 0.0150 |
| rs12153160 | | 3.822 | 0.348296 | 0.969 | 0.0169 |
| rs3843585 | | 4.062 | 0.257182 | 0.986 | 0.0124 |
| rs9787796 | | 3.133 | 0.362779 | 1.001 | 0.0180 |

**Table S2.** List of SNPs associated with ADHD (P x 10^-8^) and proxies where used

| **SNP** | **Original SNP if proxy used** | **r^2^ for proxy** | **ADHD** | | **Tobacco initiation** | |
| --- | --- | --- | --- | --- | --- | --- |
|  |  |  | **OR** | **SE** | **β** | **SE** |
| rs9677504 |  |  | 1.13202 | 0.0240 | 0.0196 | 0.0195 |
| rs4916723 |  |  | 0.91183 | 0.0158 | -0.0198 | 0.0120 |
| rs6990255 | rs74760947 | 0.95 | 1.16393 | 0.0357 | 0.0115 | 0.0272 |
| rs11591402 |  |  | 0.89987 | 0.0187 | -0.0043 | 0.0142 |
| rs1427829 |  |  | 1.08307 | 0.0155 | 0.0232 | 0.0117 |
| rs281324 |  |  | 0.92960 | 0.0154 | -0.0393 | 0.0116 |
| rs212165 | rs212178 | 0.92 | 0.88665 | 0.0231 | -0.0161 | 0.0184 |
